# High Dietary Lipid Level Is Associated with Persistent Hyperglycaemia and Downregulation of Muscle Akt-mTOR Pathway in Senegalese Sole (*Solea senegalensis*)

**DOI:** 10.1371/journal.pone.0102196

**Published:** 2014-07-18

**Authors:** Pedro Borges, Luísa M. P. Valente, Vincent Véron, Karine Dias, Stéphane Panserat, Françoise Médale

**Affiliations:** 1 CIMAR/CIIMAR, Centro Interdisciplinar de Investigação Marinha e Ambiental and ICBAS, Instituto de Ciências Biomédicas de Abel Salazar, Universidade do Porto, Porto, Portugal; 2 INRA-UR 1067 Nutrition Métabolisme Aquaculture, Pôle Hydrobiologie, Saint Pée-sur-Nivelle, France; National Institute of Nutrition, India

## Abstract

High levels of dietary lipids are incorporated in feeds for most teleost fish to promote growth and reduce nitrogen waste. However, in Senegalese sole (*Solea senegalensis*) previous studies revealed that increasing the level of dietary lipids above 8% negatively affect growth and nutrient utilization regardless of dietary protein content. It has been shown that glucose regulation and metabolism can be impaired by high dietary fat intake in mammals, but information in teleost fish is scarce. The aim of this study was to assess the possible effect of dietary lipids on glucose metabolism in Senegalese sole with special emphasis on the regulation of proteins involved in the muscle insulin-signalling pathway. Senegalese sole juveniles (29 g) were fed two isonitrogenous diets (53% dry matter) for 88 days. These two diets were one with a high lipid level (∼17%, HL) and a moderate starch content (∼14%, LC), and the other being devoid of fish oil (4% lipid, LL) and with high starch content (∼23%, HC). Surprisingly, feeding Senegalese sole the HL/LC diet resulted in prolonged hyperglycaemia, while fish fed on LL/HC diet restored basal glycaemia 2 h after feeding. The hyperglycaemic phenotype was associated with greater glucose-6-phosphatase activity (a key enzyme of hepatic glucose production) and lower citrate synthase activity in the liver, with significantly higher liver glycogen content. Sole fed on HL/LC diet also had significantly lower hexokinase activity in muscle, although hexokinase activity was low with both dietary treatments. The HL/LC diet was associated with significant reductions in muscle AKT, p70 ribosomal S6-K1 Kinase (S6K-1) and ribosomal protein S6 (S6) 2 h after feeding, suggesting down regulation of the AKT-mTOR nutrient signalling pathway in these fish. The results of this study show for the first time that high level of dietary lipids strongly affects glucose metabolism in Senegalese sole.

## Introduction

The main functions of dietary lipids are energy provision and storage in body compartments as energy reserves [1]. High levels of dietary lipids are incorporated in feeds for most teleost fish to promote growth and reduce nitrogen waste [2]. However, the Senegalese sole is a lean fish with atypical lipid metabolism, due to the limited capacity to utilize or store dietary lipids (less than 7% wet weight basis). Previous studies revealed that increasing the level of dietary lipids above 8% negatively affect growth performance and nutrient utilization, resulting in decreased protein accretion and slower growth rate [3]. It was initially hypothesized that the results obtained might be linked to the high dietary protein level incorporated into the diets (56% of dry matter - DM). However, further research demonstrated that even below the dietary protein requirement, increasing dietary lipid levels did not promote better protein retention or growth performance [4], despite the fact that *Senegalese sole* can digest and absorb dietary lipids efficiently [5]. Surprisingly, the diets with low fat content, but rich in dietary starch enhanced PFK-1 activity in the muscle [4], suggesting the potential use of carbohydrates as a non-protein energy source in this species.

There has been considerable debate over the years about the limited ability of carnivorous teleost fish to utilize dietary carbohydrates efficiently, and impaired control of plasma glucose levels, leading to glucose intolerance in such species [6]. Despite having all the metabolic pathway to utilize dietary carbohydrates [7], carnivorous fish in general cannot tolerate more than 20% of dietary carbohydrates without adverse effects on growth performance and feed utilization [8], [9]. However, carbohydrates can be used as a valuable source of energy to some extent. No effects on growth performance were reported in *Solea senegalensis* fed 11% or 19% dietary carbohydrates, regardless of the nature of carbohydrates (raw or extruded starch) or dietary lipid level (11 and 21%) [10]. It was recently suggested [11] that protein content could be reduced from 55% to 45% by increasing dietary starch from 8.6% to 19.6%, indicating efficient use of carbohydrates as a non-protein energy source in this species. However, the mechanisms remain to be explained that allow *Solea senegalensis*, a lean fish with low capacity to utilize or store dietary lipids, to achieve better growth performance when fed diets with low fat (4% DM) and energy content compared to fish fed diet with high lipid content (12–20% DM) [3], [4]. We therefore, hypothesized that the observed growth impairment when sole were fed diets with high lipid content might be linked to the lean nature of this species, and might result from a metabolic disorder promoted by interaction between lipids and carbohydrates, as reported in higher vertebrates [12], [13]. Most studies in fish species such as trouthave been focused on the relationship between dietary carbohydrates and proteins in the development of impaired glucose regulation [14], [15]. However, the effects of dietary lipid levels on this phenomenon have not been investigated in detail and no results are available regarding lean fish.

Panserat et al. [16] reported ten years ago that high lipid diets induced glucose-6-phosphatase expression, contributing to hyperglycaemia. Figueiredo et al. [17] recently reported that diets with high lipid/high carbohydrate content resulted in prolonged hyperglycaemia and exogenous insulin resistance in rainbow trout, similar to that observed in pre-diabetic mammalian subjects. These authors also observed a significant reduction of insulin receptor substrate 1 (IRS1) protein content in muscle of trout fed on the high lipid- diet. IRS proteins are considered to be key components in the insulin-signalling cascade [18]. Once activated by insulin binding, insulin receptor (IR), a tyrosine kinase membrane receptor, phosphorylate IRS, which in turn initiates numerous downstream cellular responses through several molecules. One such key molecule in the signalling cascade is phosphatidylinositol 3-kinase (PI3K) that subsequently phosphorylates a number of downstream proteins including AKT, also known as protein kinase B [19]. The cascade IRS-PI3K-AKT can phosphorylate mTOR which activates downstream proteins like ribosomal S6 kinase protein (S6K1) and finally, leads to the regulation of genes involved in intermediary metabolism, protein accretion and cell growth, among other functions [20], [21], [22]. The AKT-mTOR pathway can be activated by either amino-acids or insulin in rainbow trout as in mammals and is sensitive to the dietary protein to carbohydrate ratio. As such, it is considered as a major nutrient signalling pathway in fish [20], [23], [24]. However, it has never been investigated in Senegalese sole. Although the precise involvement of insulin-signalling defects in the development of insulin resistance remains unclear even in mammals, a reduction of the IRS1 protein content in insulin-sensitive tissues, like skeletal muscle, has been proposed as one of the mechanisms inducing insulin resistance in mammals [23] and, more recently, in trout [24].

Although there is no single metabolic aberration that precedes hyperglycaemia, the current epidemics of diabetes and obesity are seemingly related [25] and several studies have been carried out establishing that high fat intake as one of the causes of the development of this condition in mammals [26]–[31]. It was initially hypothesized that hyperglycaemia resulting from high carbohydrate intake by carnivorous fish was due to insufficient insulin secretion [32]. However, it was later observed that plasma insulin levels were equal to or higher than mammals [33]. The other possible causes suggested have been the reduced number of muscle insulin receptors [34] and the low affinity of glucose to its transporters [35], as well as a lack of capacity to downregulate liver gluconeogenic pathways [16], which constitutes one step to achieving normal glucose homeostasis [36], [37].

The purpose of this study was to analyse the consequences of two isoproteic diets with different lipid/carbohydrate ratios on plasma metabolites and glucose metabolism of the Sole. Juveniles of *Senegalese sole* were fed one of two isonitrogenous diets: the HL/LC diet had a high lipid level and moderate carbohydrate content (∼17% lipids and ∼14% starch DM basis) while the LL/HC diet had no addition of fish oil however contained high level of dietary carbohydrates (∼4% lipids and ∼23% starch DM basis). At the end of the trial, plasma glucose, triglyceride and lactate levels were analyzed at different time intervals. Glycogen content and key glycolytic enzyme activity were measured in the liver and muscle. Muscle proteins involved in the AKT-mTOR pathway (the major nutrient signalling pathway) like insulin receptor (IR), AKT, S6K-1 and S6 were also assayed to assess the impact of dietary lipid to carbohydrate ratios.

## Materials and Methods

### Ethics Statement

Experiments were conducted by trained scientists (following FELASA category C recommendations) and carried out in accordance with the clear boundaries of EU legal frameworks, specifically those related to the protection of animals used for scientific purposes (i.e. Directive 2010/63/EU) and under the Portuguese legislation regarding the protection of animals used for scientific purposes (Law N° 113/2013). The study was performed at the experimental facilities of CIIMAR, Porto, Portugal, certified for animal experiments by Direcção Geral de Alimentação e Veterinária, which is the competent authority. All procedures used in this study were approved by the ethics committee of CIIMAR. The experimental diets met nutritional requirements of flatfish according to NRC (2011) and fish were sedated with an anaesthetic before handling.

### Experimental diets

Two experimental diets were formulated to contain 54% protein (DM basis) and two different levels of lipids (4 and 17% DM) and starch (14 and 23% DM) (Table 1). Increased lipid level was achieved by adding fish oil and lowering the amount of wheat meal, so that the diet with 17% lipids had lower starch content (14% DM) than the diet with 4% lipids (23% DM). Ingredients and chemical composition are presented in Table 1. All ingredients were finely ground, mixed and dry-pelleted (2.0 mm diameter) without steaming, using a laboratory pelleting machine (C-300 model; California Pellet Mill, San Francisco, CA, USA).

**Table 1:** Ingredients and composition of the experimental diets with different lipid/carbohydrate levels.

	Dietary treatments	
	LL/HC	HL/LC
**Ingredients (g/100 g)**		
Fishmeal LT	30.00	30.00
CPSP G	8.50	8.50
Soybean meal 48	12.50	10.60
Corn gluten	9.00	9.00
Wheat meal	27.20	14.10
Wheat gluten	11.80	14.80
Fish oil	0	12.00
Choline chloride	0.10	0.10
Lutavit C35	0.03	0.03
Lutavit E50	0.05	0.05
Vit^1^ & Min^2^ Mix	0.25	0.25
Betaine	0.07	0.07
DCP	0.50	0.50
**Composition (g/100 g of dry matter)**		
Dry matter	91.42	92.68
Ash	7.64	7.37
Crude protein	54.41	53.87
Crude fat	4.74	17.34
Starch	23.29	14.14
Gross Energy (kJ/g)	21.00	23.75

LT, low temperature; CPSP G, fish soluble protein concentrate (hydrolysed fishmeal); Lutavit C35, vitamin C; Lutavit E50, vitamin E; DCP, dibasic calcium phosphate.

1 Vitamins (per kg diet): vitamin A, 10000 IU; vitamin D3, 2125 IU; vitamin K3, 12.5 mg; vitamin B12, 0·025 mg; vitamin B1, 10 mg; vitamin B2, 25 mg; vitamin B6, 12.5 mg; folic acid, 12.5 mg; biotin, 0.86 mg; inositol, 300 mg; nicotinic acid, 85 mg; pantothenic acid, 37.50 mg.

2 Minerals (per kg diet): Mn (manganese oxide) 25 mg; I (potassium iodide) 1.88 mg; Cu (copper sulfate) 6.25 mg; Co (cobalt sulfate) 0.13 mg; Zn (zinc oxide) 37.5 mg; Se (sodium selenite) 0.31 mg; Fe (iron sulfate) 75 mg.

### Experimental conditions

Senegalese sole (*Solea senegalensis*) juveniles were obtained from a commercial fish farm (Coelho & Castro, Portugal). After arrival at the experimental unit, fish were acclimatized to the new facilities for two weeks. Homogeneous groups of 20 fish (average initial body weight 29±1.7 g; initial density 3.4 kg/m^2^) were distributed into six 45 L white fibreglass tanks (50 cm×35 cm) and each diet was tested in triplicate, with 60 individuals in each dietary treatment. To ensure homogeneity of the groups, fish were selected according to their weight. Tanks were randomly assigned to each treatment. Each tank was supplied with filtered, heated (20±1°C) salt water (30‰), at a flow rate of 1.5 L min^−1^. Water parameters (temperature, dissolved O_2_, salinity, pH and nitrogenous compounds) were monitored during the entire trial and maintained at levels within limits recommended for marine species. Fish were exposed to an artificial photoperiod of 12 h light. At the beginning and end of the experiment, individual weights and lengths of fish were recorded. Fish were fed *ad libitum* six to eight meals a day (24 h) by automatic feeders over a period of 88 days. All tanks were monitored daily and feed distribution adjusted based on feed losses in each tank [3].

Ten fish from the initial stock and three fish per tank, at the end of the trial were sampled and stored at −20° for subsequent whole body analysis. Three days before sampling, feed distribution was reduced to two meals a day to satiation (8 a.m. and 20 p.m.) to ensure that all fish would have eaten on the sampling day and that plasma glucose levels could be assessed up to 16 hours after feeding, without being affected by the previous meal. The last meal before sampling was delivered in the morning (8 a.m.). Three fish per tank were then sampled at different time intervals after the meal (0.5, 1, 2, 5, 9, 12 and 16 h after feeding). All fish were randomly selected for subsequent analysis. Fish were anaesthetized with MS-222 (200 mg/L) to achieve stage II of anaesthesia and only sampled when they did not respond to stimulus (∼3 min to be completely anesthetized). Blood samples were taken from the caudal vein, using syringes (1 ml) containing 20 µL EDTA 2% and plasma was obtained after centrifugation (5000×g for 5 min at 4°C) and stored at −80°C pending analysis of glucose, lactate and triglyceride levels. After blood collection, fish were sacrificed by decapitation and intestinal contents were examined to check whether they had eaten. Whole livers and samples from the anterior part of the white muscle were collected five hours after feeding to determine glycogen content. For enzymatic assays, liver and muscle were collected 16 h after feeding to assess the possible effect of the dietary treatments. For Western Blot analysis, muscle was collected both at 2 h, to assess AKT-mTOR signal transduction, when it peaks (short term regulation) [24], and also 16 h after feeding, when enzyme activities were also measured. All samples were frozen in liquid nitrogen immediately after dissection and kept at −80°C.

### Analytical methods

Whole fish from each tank were ground and pooled, and moisture content was measured (105°C for 24 h). Fish were subsequently freeze-dried, before further analysis. Feed and whole body samples were analyzed for dry matter (105°C for 24 h), ash by combustion in a muffle furnace (Nabertherm L9/11/B170; Bremen, Germany; 550°C for 6 h), crude protein by automatic flash combustion (Leco FP-528, Leco, St. Joseph, USA; N×6.25), lipid content by petroleum ether extraction, using a Soxtherm Multistat/SX PC (Gerhardt, Königswinter, Germany; 150°C) (feed and whole body samples only), and gross energy in an adiabatic bomb calorimeter (Werke C2000; IKA, Staufen, Germany).

Liver and muscle glycogen levels were determined following the method of Keppler et al. [38]. Hexokinase (HK; EC 2.7.1.1) and glucokinase (GK; EC 2.7.1.2) activities were determined as previously described [16] and PFK-1 (EC 2.7.1.11) activity was assayed as described in Borges et al. [4]. To assess citrate synthase and glucose-6-phophatase activities, tissues were homogenized by ultrasonic disruption in 9 vol of ice-cold buffer containing 50 mmol/L Tris (pH 7.6), 5 mmol/l EDTA, 2 mmol/L 1,4-dithiothreitol, and a protease inhibitor cocktail (Sigma, St. Louis, MO; P-2714). The homogenate was centrifuged, and the supernatant was used immediately for enzyme assays. Citrate synthase (CS; EC 4.1.3.7) was measured according to Singer et al. [39] by following the reduction of DTNB at 412 nm. G6Pase was measured according to Alegre *et al*. [40], monitoring the increase in absorbance (NADH production), using glucose dehydrogenase (Sigma) in excess, as the coupling enzyme. One unit of enzyme activity was defined as the amount of enzyme that catalyzed the hydrolysis of 1 µmol of substrate per minute under the specified conditions. Enzyme activity was expressed per mg of soluble protein. Protein concentration was measured according to Bradford's method [41], using a protein assay kit (Bio Rad, München, Germany) with bovine serum albumin as standard.

For Western blotting, individual muscle samples (300 mg) were homogenized on ice with an Ultraturrax homogenizer in 9 vol of buffer containing 150 mmol/L NaCl, 10 mmol/L Tris, 1 mmol/L EGTA, 1 mmol/L EDTA (pH 7.4), 100 mmol/L sodium fluoride, 4 mmol/L sodium pyrophosphate, 2 mmol/L sodium orthovanadate, 1% Triton X-100, 0.5% NP-40-IGEPAL and a protease inhibitor cocktail (Roche, Basel, Switzerland). Homogenates were centrifuged for 15 min at 12,000g and the resulting supernatant were stored at −80°C. Protein concentrations were determined, using the Bio-Rad protein assay kit (BIO-RAD, Hercules, CA, USA). Protein lysates (40 µg of protein) were subjected to SDS–PAGE and Western blotting using anti-IR, anti-phospho-AKT, anti-phospho-S6K1 and anti-phospho-S6. After washing, membranes were incubated with an IRDye infrared secondary antibody (Li-COR Inc. Biotechnology, Lincoln, NE, USA). Bands were visualized by Infrared Fluorescence, using the Odyssey Imaging System (Li-COR Inc. Biotechnology, Lincoln, NE, USA) and quantified by Odyssey infrared imaging system software (Application Software, version 1.2). Gels were then stripped and total protein forms and B tubulin were quantified to normalize protein expression.

### Statistical analysis

Statistical analyses followed the methods outlined by Zar [42]. All data were tested for homogeneity of variances by Leven's tests, and then submitted to one-way ANOVA at each sampling point or to two-way ANOVA with time and diet as independent variables. When diet x time interactions were significant (P≤0·05), individual means were compared using Tukey's test. For growth performance data (Table 2) the experimental unit was the tank, after pooling 3 fish (3/20), and for the remaining analysis, the experimental unit was individual fish. All data were analysed using IBM SPSS statistics version 19 (IBM Corp., New York, *USA*). Differences were considered significant when P<0.05.

**Table 2:** Effects of LL/HC or HL/LC diet on Senegalese sole growth, intake and nutrient gain and whole body composition.

			Dietary treatments							
			LL/HC				HL/LC			
**Growth**										
Initial body weight (g)			29.21	±	0.74		29.04	±	0.83	
Weight Gain (g)			29.76	±	1.63		26.40	±	2.10	
Daily Growth Index (DGI)			0.92	±	0.05		0.84	±	0.06	
Feed efficiency (FE)			0.75	±	0.06		0.69	±	0.05	
**Intake (g or kj/ABW Kg/day)**										
	Protein		5.56	±	0.40		5.54	±	0.45	
	Lipids		0.48	±	0.03	b	1.78	±	0.14	a
	Starch		2.38	±	0.17	a	1.45	±	0.12	b
	Energy (kj)		214.63	±	15.34		240.06	±	19.85	
**Gain (g/Kg/day)**										
	Protein		1.67	±	0.04	a	1.09	±	0.08	b
	Lipids		0.32	±	0.14		0.50	±	0.10	
**Whole body composition (%WW)**										
	Protein		19.22	±	0.27	a	18.16	±	0.35	b
	Lipids		4.80	±	1.02	b	7.18	±	0.88	a

Results are expressed as means ± SD (n = 3). Data were submitted to a one way ANOVA.

a-b Mean values within a row with different superscript letters were significantly different (P0·05).

DGI, daily growth index; FE, feed efficiency; ABW, average body weight.

DGI = 100×((final body weight)1/3 - (initial body weight)1/3)/days.

FE = weight gain/dry feed intake.Nutrient intake = nutrient intake/average body weight ((initial body weight+final body weight)/2)/days.

WW =  wet weight

## Results

Data on weight gain, feed efficiency, nutrient intake and gain and whole body composition of *Senegalese sole* fed the two diets for 88 days are presented in Table 2. Weight gain and daily growth index were similar with both dietary treatments, although sole fed on LL/HC diet reached a slightly higher final weight than those fed on HL/LC diet. Protein and energy intake were similar between treatments however lipid and starch intake were significantly different due to difference in feed formulae (p<0.05). As expected, fish fed on HL/LC diet had a higher lipid intake than those fed on LL/HC diet (1.78 vs 0.48), whereas the opposite was recorded for starch intake (HL/LC = 1.45 and LL/HC = 2.38). Protein gain was significantly higher in fish fed the LL/HC diet and no significant differences were recorded for lipid gain, although the value was slightly higher in fish fed the HL/LC diet. In accordance with protein and lipid gain, whole body protein was higher with the LL/HC diet compared to the HL/LC diet and the inverse was recorded for whole body lipid gain.

**Figure 1:**
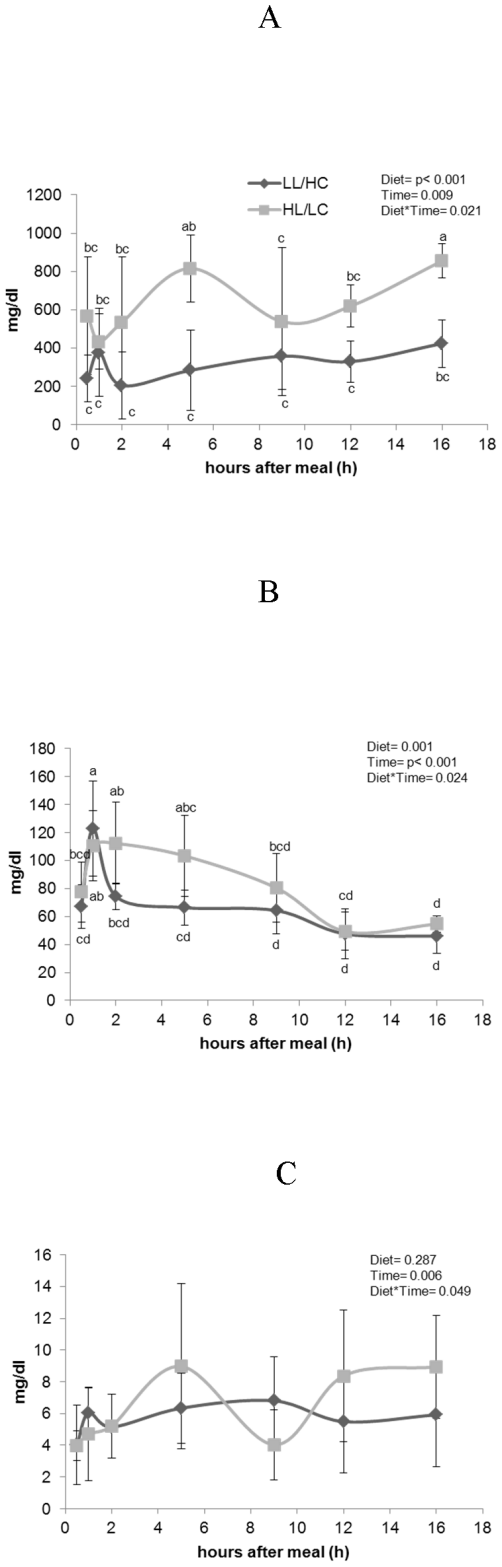
Postprandial plasma triglycerides (A), glucose (B) and lactate (C) of fish fed LL/HC or HL/LC diet (n = 9). Data were submitted to a two way ANOVA.

Two-way ANOVA was conducted to examine the effects of dietary treatment and postprandial time on plasma triglyceride (Fig. 1A), glucose (Fig. 1B) and lactate (Fig. 1C) concentrations. In general, there was a significant interaction between the effects of dietary treatment and postprandial time for all three parameters analysed. Plasma triglyceride levels were higher in the HL/LC than in LL/HC treatment, although diet-induced differences were only significant at 5 and 16 h after the meal. Glucose plasma levels peaked between 1 and 2 hours after feeding. The HL/LC diet resulted in persistent elevated plasma glucose levels from 1 h until 5 h after feeding, while glucose levels decreased one hour after reaching the peak in fish fed the LL/HC diet, despite larger carbohydrate intake. Despite the interaction observed, plasma lactate levels did not present any differences between treatments due to wide individual variations.

**Figure 2:**
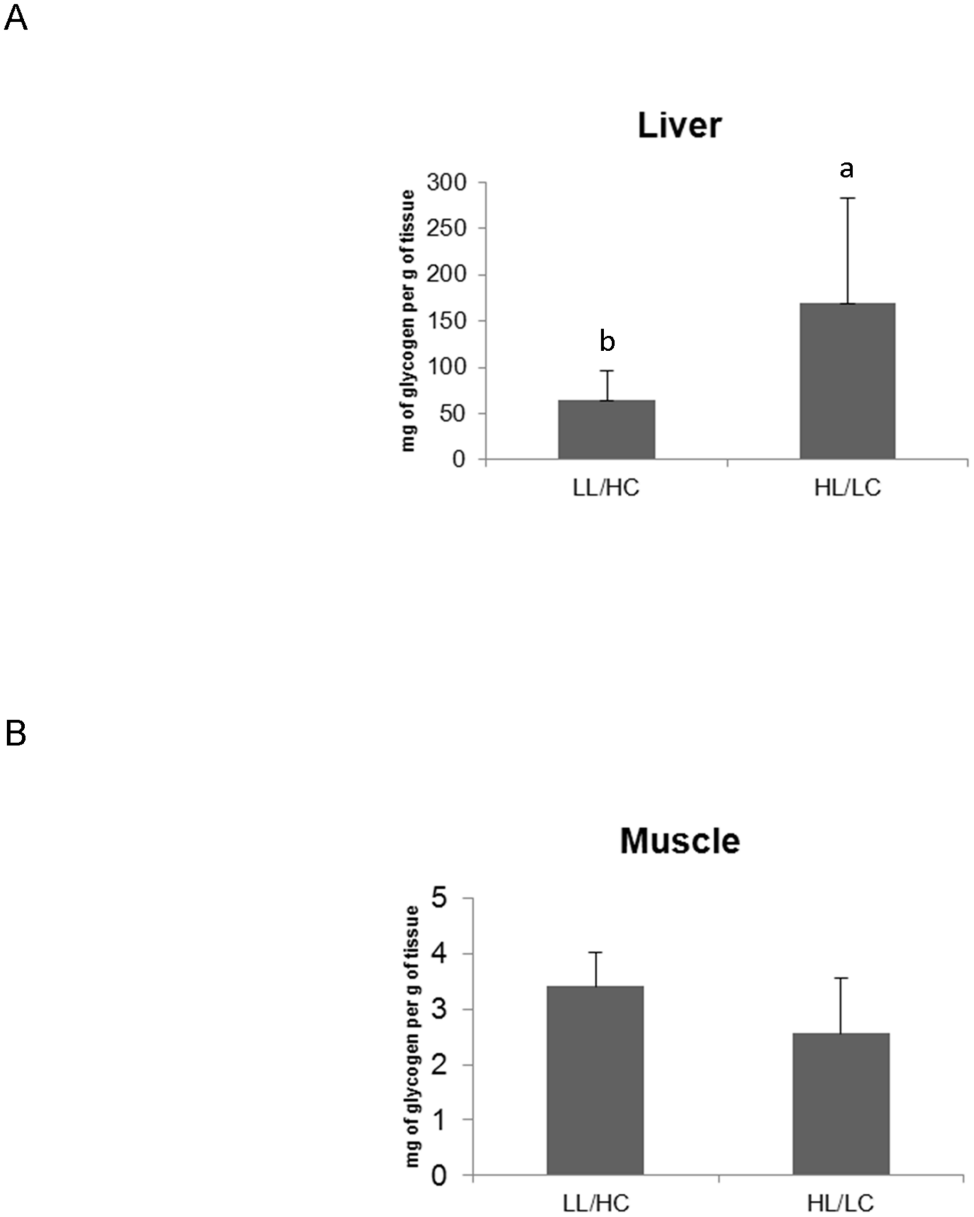
Liver (A) and muscle (B) glycogen of fish fed LL/HC or HL/LC diet (n = 9). Data were submitted to a one way ANOVA.

Liver glycogen content (Fig. 2A) was significantly higher in fish fed on HL/LC diet, while glycogen levels in the muscle (Fig. 2B) were not affected by dietary treatments. GK and HK activity in the liver (Table 3) were comparable, irrespective of dietary treatment. On the other hand, G6Pase activity was significantly higher in fish fed the HL/LC diet compared to those fed the LL/HC diet, whereas CS was down-regulated. Muscle-HK activity was significantly higher in fish fed on LL/HC diet compared to those fed on HL/LC diet. Muscle PFK and CS activities were comparable between treatments.

**Table 3:** Activities (mU/mg of protein) of liver Hexokinase (HK), Glucokinase (GK), Glucose-6-phosphatase (G6Pase) and Citrate synthase (CS) and muscle HK, phosphofructokinase-1 (PFK-1) and CS 16 h after feeding LL/HC or HL/LC diet.

		LL/HC				HL/LC			
**Liver**	**HK**	1.2	±	0.89		2.56	±	1.92	
	**GK**	6.98	±	4.61		10.41	±	2.99	
	**G6Pase**	39.79	±	7.66	b	50.17	±	3.94	a
	**CS**	26.77	±	15.56	b	13.99	±	5.07	a
									
**Muscle**	**HK**	1.98	±	0.7	a	1.4	±	0.36	b
	**PFK-1**	539.95	±	378.06		687	±	191	
	**CS**	17.783	±	4.7245		15.99	±	4.38	

Results are expressed as means ± SD (n = 9). Data were submitted to a one way ANOVA.a-b Mean values within a row with different superscript letters were significantly different (P,0·05).

**Figure 3:**
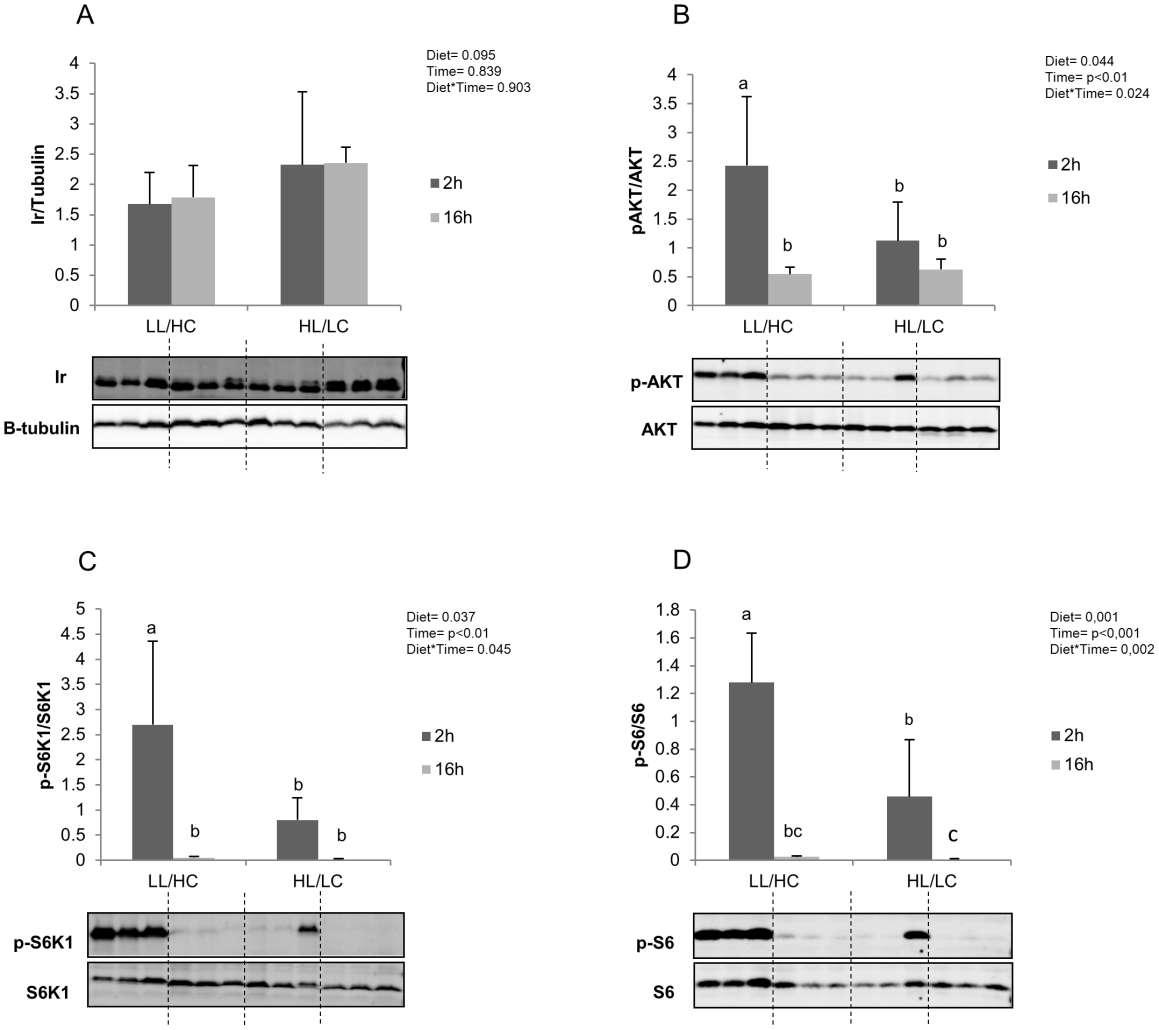
Insulin receptor (IR-A) levels and Akt (B), S6K1 (C) and S6 (D) phosphorylation status in muscle of Senegalese sole fed LL/HC or HL/LC diet (n = 6). Results are expressed as the ratio between total protein and reference protein (β-tubulin) for IR and ratio between phosphorylated form and total form for the remaining proteins. Representative blots are shown. Data were submitted to a two way ANOVA. Gels were loaded with 40 µg total protein per lane.

No significant differences were found with regards to muscle IR content (Fig. 3A), irrespective of dietary treatment or sampling time. There was a significant interaction between the effects of dietary treatment and postprandial time on muscle AKT. AKT phosphorylation status (Fig. 3B) was increased approximately 2-3 fold, 2 h after feeding on LL/HC diet, although no differences were noted in the total AKT content. Sixteen hours after feeding, AKT phosphorylation status on LL/HC diet decreased to values close to those with the HL/LC diet. For both S6K1 (Fig. 3C) and S6 (Fig. 3D), there was a significant interaction between the effects of dietary treatment and postprandial time. The results clearly demonstrated that this pathway was more active 2 h after feeding than sixteen hours after feeding, and phosphorylation status of these proteins clearly increased 2 h after feeding the LL/HC diet.

## Discussion

The decrease in dietary lipid to carbohydrate ratio (LL/HC diet) promoted higher protein gain and a slight increase in growth, when compared to the high fat to low carbohydrate ratio (HL/LC diet). From our first report [3] until now, several studies have reported lower or similar growth performance in *Senegalese sole* juveniles fed on high lipid diets, compared to low lipid diets, with no advantage to growth performance following the inclusion of fat as a non-protein energy source [4], [10], [43], [44]. By contrast, in other carnivorous species, high fat diets have been reported to promote better growth [45]–[48], although mostly due to increased fat deposition. In the sole, the HL/LC diet also increased whole body fat content, however decreased whole body protein and protein gain, as in previous studies, where similar lipid levels were tested [3], [4], [43]. Plasma triglyceride levels reflected the difference in lipid content of both diets. Five hours after the meal, the HL/LC diet resulted in higher plasma triglyceride level than the LL/HC diet, which seems to be the peak of lipid absorption in this species [5]. Compared to lipids, the use of carbohydrates, as energy source in teleost fish is limited [8], [49], [50]. It was recently reported that dietary protein could be reduced from 55% to 45%, through an increase in starch level in diets with high lipid levels (16%), suggesting good use of carbohydrates in this species [11]. In the present study, the LL/HC diet led to better overall growth performance. Despite having a starch level slightly higher than recommended for carnivorous species (less than 20%), fish fed this diet re-established plasma glucose levels two hours after feeding. On the other hand, the HL/LC diet resulted in typical hyperglycaemic phenotype of fish fed on carbohydrates, despite supplying lower amount of starch than the LL/HC diet. Increasing lipid content to promote a protein sparing effect, has become a common practice in fish nutrition. Although there are a few studies linking dietary fat intake to carbohydrate metabolism in fish species, some authors have reported that diets with high lipid content can also induce hyperglycaemia [17], [51]–[53].

The observed development of hyperglycaemic phenotype in fish fed on HL/LC diet could be due to dietary lipid content, as these fish regulated plasma glucose homeostasis less efficiently than fish fed the LL/HC diet (higher starch and lower lipid intakes). These results are in general accordance with the observations in higher mammals, where higher dietary fat intake and higher plasma triglyceride levels result in impaired glucose tolerance [27], [54], [55]. In addition to the higher plasma glucose levels, HL/LC diet affected liver glycogen content. Glycogen content in mammalian liver can vary as much as two-fold during the day, increasing from breakfast (the lowest point) until 4–5 hours after dinner [56]. In the present study, at 5 hours after the meal, the HL/LC diet resulted in increased liver glycogen content associated with hyperglycaemia, as also previously reported in rainbow trout [16], [17] and sea bream [57]. The pathways involved in this phenomenon do not seem to be regulated in the same way as in higher vertebrates, since in the latter hyperglycaemia is accompanied by lower glycogen levels [58].

Regulation of gluconeogenesis (endogenous glucose production) and glycolysis is coordinated, so that when one of the pathways is active the other is relatively inactive. The liver participates in blood glucose homeostasis by regulating glucose storage and utilization (energy production) and gluconeogenesis according to metabolic state. When glucose is taken up, it is phosphorylated and enters either the glycolysis or glycogen synthesis pathway. In *Senegalese sole*, liver glucokinase activity, the first step of glycolysis, was similar with both treatments, whereas G6Pase (last step of glucose production) was up-regulated in fish fed HL/LC diet, contributing possibly to the hyperglycaemia. Gluconeogenesis in *Senegalese sole* fed on high protein/low starch diet was higher compared to a low protein/high starch diet and this was attributed to increased protein supply throught diet [11]. However, in the latter study, diets were isolipidic. In studies on rainbow trout wherein a possible interaction between dietary lipids and glucose utilization was evaluated, high lipid-diets did not cause such a response with regard to G6Pase [16], [17]. In contrast to the results obtained in the present study, protein intake was significantly greater with low fat diets, probably inducing G6Pase up-regulation [15].

Moreover, the activity of mitochondrial citrate synthase was lower in the HL/LC group than in the LL/HC group, suggesting that oxidative phosphorylation in the liver was less active in fish fed on HL/LC diet. It was observed that liver citrate synthase and oxygen consumption were comparable in obese and lean zucker rats [59]. However, recent findings have shown that impaired oxidative phosphorylation mechanisms in muscle mitochondria might be responsible for insulin resistance and resultant hyperglycaemia [60]–[62].

In brown trout (S*almo truta*), white muscle is the main tissue responsible for glucose disposal after carbohydrate intake, although only 50% of glucose is taken up 10 h after feeding [63]. In contrast, mammals take only two hours after feeding to uptake 85% of the glucose absorbed [64]. Nonetheless, it was observed in brown trout that most of the glucose taken up was phosphorylated despite low hexokinase activity. In the present study, hexokinase was up-regulated in sole fed on LL/HC diet, but no significant diet-induced effects were found for PFK-1 or citrate synthase. Borges et al. [4] reported that PFK-1 activity, one of the early rate-limiting steps of glycolysis [65], [66], was up-regulated in fish fed diets with low fat level in combination with low protein and high starch content. The disparity in the results can be attributed to the differences in the starch contents (lower in the present study) and sampling time (6 h *vs* 16 h in the present study), resulting in almost 50% reduction in the activity of this enzyme (∼1000 vs ∼550 mU/mg protein). In contrast to liver, muscle citrate synthase activity was not affected by the dietary treatments. As stated earlier, several studies have reported mitochondrial oxidative dysfunction in subjects with impaired glucose tolerance and insulin resistance, although down-regulation of citrate synthase activity was only detected in *in vitro* myocyte culture [61].

Despite the lack of significant difference in muscle glycogen content, the HL/LC diet reduced the levels of phosphorylated AKT, S6K1 and S6. It is well known that AKT, S6K1 and S6 can be activated by amino acids [21], [24], [67]. However, in the present study protein intake was similar with both diets, suggesting that amino acids are not responsible for the depression of the insulin signalling pathway recorded in the fish fed the HL/LC diet. The insulin receptor (IR) level was similar with both diets, but the diet-induced changes in downstream events two hours after feeding suggested a different level of interaction between IR and its ligand (insulin or insulin growth factor 1). There are several similarities in insulin receptors of fish and mammals [34], and similar responses are triggered upon ligand-receptor interaction [68]–[71]. The receptor-ligand interaction is the beginning of a network of possible responses that depend on the crosstalk between pathways. According to Taniguchi et al. [72], AKT is involved at a critical stage in the insulin signalling pathway. After upstream activation (insulin receptor (IR)/insulin receptor substrate), AKT phosphorylation can initiate the signalling of the family of proteins involved in cytoskeletal re-organization that is required for the translocation of glucose transporter GLUT4, promoting greater glucose uptake. Although not conclusive, the impaired glycaemic regulation observed in HL/LC group may be due to defective signalling of skeletal muscle insulin, explaining to some extent the observed lower protein gain in fish fed HL/LC diet.

On the contrary, the higher level of AKT phosphorylation observed with LL/HC diet compared to HL/LC diet led to downstream activation of S6K1 and S6, the major indicators of the mTOR (nutrient) signalling cascade, which is involved in growth and nutrient sensing. In *Senegalese sole*, apart from IGF findings [73], there are no studies relating insulin and carbohydrate utilization. Although it has not been possible to accurately quantify circulating insulin levels in the sole, we found that the muscle levels of phosphorylated AKT, S6K1 and S6 were increased in the group fed on high carbohydrates diet. Given the observation that plasma glucose levels were normalized to the basal level along with greater protein gain it appears that Senegalese sole seemed to cope well with a diet with high starch level and low lipid level.

Previous studies in *Senegalese sole* have demonstrated growth impairment and lower protein accretion in fish fed on high fat diets. Our study sheds some light on this subject, by demonstrating a lipid/carbohydrate interaction in the glucose regulatory pathway. In conclusion, HL/LC diet affected glucose metabolism leading to prolonged hyperglycaemia, probably by increasing endogenous glucose production. Data obtained from muscle support the theory of a possible insulin resistance state as levels of several major proteins involved in the insulin and nutrient signalling pathway were reduced in fish fed on high fat diet. Results suggested that starch may be a valuable source of energy in Senegalese sole diets.
